# Factor Structure of Social Cognition in Schizophrenia: Is Empathy Preserved?

**DOI:** 10.1155/2013/409205

**Published:** 2013-12-15

**Authors:** Silvia Corbera, Bruce E. Wexler, Satoru Ikezawa, Morris D. Bell

**Affiliations:** ^1^Connecticut Mental Health Center, Department of Psychiatry, Yale University School of Medicine, New Haven, CT 06508, USA; ^2^Olin Neuropsychiatry Research Center, Institute of Living, Hartford Hospital, 200 Retreat Avenue, Hartford, CT 06106, USA; ^3^Division of Neuropsychiatry, Yowa Hospital, 3-5-1 Kamigoto, Yonago, Tottori 6830841, Japan; ^4^VA Connecticut Healthcare System, Psychology Service 116B, VACHS, 950 Campbell Avenue, West Haven, CT 06516, USA

## Abstract

Social cognitive impairments are core features of schizophrenia and are closely associated with poor functional outcome. This study sought to identify specific aspects of social cognition and their relationships to measures of social function, quality of life, and neurocognition. Principal component analysis was performed using social cognitive measures in patients with schizophrenia and healthy matched controls and revealed three factors: Interpersonal Discomfort, Basic Social Cognition, and Empathy. Patients had higher scores on Interpersonal Discomfort and lower scores on Basic Social Cognition than controls, but the two groups were the same on Empathy. Lower social performance was significantly correlated with poor Basic Social Cognition in patients and with high Interpersonal Discomfort in controls. While neurocognition was significantly associated with Basic Social Cognition in both groups, it was not associated with Empathy. Social cognitive interventions should emphasize improving basic social cognitive processing deficits, managing Interpersonal Discomfort, and utilizing preserved capacity for empathy as a potential strength in social interactions.

## 1. Introduction

Patients with schizophrenia have poor social cognitive skills, and these deficits greatly impact their daily functioning [1]. Additionally, social cognition has been found to mediate the relationship between neurocognition and social functioning [2, 3]. Treatment interventions have proliferated to address deficits in social cognition as ways to improve social functioning and several meta-analyses strongly support the efficacy of the interventions [4–6]. However, social cognition and social function are two broad multidimensional constructs and relatively little is known about the relationships among more specific aspects of each, either within or across the two broader spheres, or what aspects of each are most responsive to current treatments. One recent meta-analysis by Kurtz and Richardson [6] compared treatment effects on various measures of social cognition and found the greatest effects on facial affect recognition and lesser effects on measures of other aspects of social cognition. More work is needed to clarify which social cognitive constructs most directly influence social functioning [3] and which are more or less amenable to existing treatments. Understanding the social cognitive constructs that most relate to social functioning would help identify targets to treat in social cognitive interventions.

Some studies have attempted to study the relationships between selected social cognitive constructs. Langdon and colleagues [7] found that patients with schizophrenia show a general deficit in emotion attribution and Theory of Mind (ToM), proposing a general impairment in ascribing beliefs, intentions, and emotions to others. Yet, there is increasing evidence that social cognitive assessments employed in schizophrenia research reflect the presence of separable factors [2, 8, 9].

Different social cognitive domains may contribute to distinct features of functioning. For example, in a recent meta-analysis, Fett and colleagues [10] found that social cognition was more strongly associated with community functioning (accounted for 16% of the variance) than neurocognition (accounted for 6% of the variance), with the strongest associations being with the domain of ToM, followed by social perception, and knowledge and emotional perception and processing. Also, in a recent review, Schmidt and colleagues [3] found that the mediating role of social cognition between neurocognition and functional outcome depended on which social cognitive domain was employed. Specifically, they found that social knowledge (mean effect size of 0.28) and social perception (mean effect size of 0.21) produced the largest effect sizes for explaining the mediation effect between neurocognition and functional outcome. These findings suggest that not all the social cognitive domains contribute equally to social and real life functioning. A basic step in developing a model of how social cognition influences function in schizophrenia is to explore the underlying factor structure of social cognition in schizophrenia using a range of instruments that may represent distinct domains.

A recent systematic review on the studies that use factor analysis to examine social cognition and neurocognition reported a lack of consistency regarding the factor structure of social cognition in schizophrenia [11]. The diversity and nature of the measures used in these studies seemed to play a major role in this inconsistency. Apparently social cognition emerged as multifactorial when the studies used a wide variety of comprehensive measures to target the full spectrum of social cognition. In contrast, studies that employed just a few measures demonstrated a single or binary factor structure [11].

In one recent study, Mancuso and colleagues [9] attempted to examine such structure and relate it to social functioning. The authors applied exploratory factor analysis to five social cognition tasks that targeted the domains of processing of emotion-related stimuli, social perception, attributional style, and Theory of Mind, in 84 patients with schizophrenia. They found a three-factor-structure solution which they named (1) Hostile Attributional Style, (2) lower-level social cue detection, and (3) higher-level inferential and regulatory processes. Factor 1 correlated with clinical symptoms and not with functional outcome, while factors 2 and 3 were related to both functional capacity and real-world functioning but not with symptomatology. None of the three factors correlated with negative symptoms. The authors concluded that social cognition should be regarded as a multidimensional hierarchical construct organized into information processing levels [9]. While this was an important step in advancing this line of investigation, further research is needed to confirm and complement these factors in regards to the underlying structure of social cognition in schizophrenia.

It was for this reason that NIMH recently supported a major research effort, called the Social Cognition Psychometric Evaluation (SCOPE), directed by Philip Harvey. This initiative began with a Rand panel process to select a range of social cognitive measures for reliability and construct validity to represent various domains based on expert opinion. Results of the first stage of this initiative were recently presented at an international conference [12]. Notably, after considerable debate, the experts excluded empathy as a domain for SCOPE. The SCOPE panel made this decision mostly because there was inadequate research literature on empathy and schizophrenia. In part, the present study is an effort to fill this important gap in our knowledge base.

The present study was begun three years before the SCOPE initiative, but unlike SCOPE, we included a measure of empathy. Hence this paper is an effort to fill this gap in the literature. We did so because empathy is a fundamental social cognitive concept in social neuroscience and has been of increasing interest to the schizophrenia field, with some regarding it as one of the most important social constructs to study in schizophrenia [12]. Indeed, a recently published study found that impairments in empathy were related to poor functioning in schizophrenia [13].

Additionally, differently from the study of Mancuso and colleagues [9] we included social cognitive measures that capture emotional perception and emotional experience. A number of studies have revealed defective emotional perception in patients [14–16]. However, emotional experience appears to be preserved in patients with schizophrenia [17–20]. The distinction of both concepts is important from the affective neuroscience perspective since both constructs are measured by different tasks and recruit different brain regions [18].

Therefore, the first goal of the current study was to determine the underlying relationship between measures of social cognition through principal components analysis in patients with schizophrenia and healthy matched controls. For that purpose, we employed three social cognitive measures that assess emotional perception and processing (Facial Affect Recognition, Theory of Mind, and Social Attribution) and two measures of emotional experience (Empathy and Self-Experience of Relatedness). Evidence has shown that empathy is comprised of two distinct systems: cognitive and emotional empathy [21]. For that purpose we included two subscales targeting each system. Regarding the Self-Experience of Relatedness construct, we selected measures that previous studies have shown distinguish schizophrenia from other clinical samples and discriminate various patterns of relatedness within schizophrenia samples [2, 22, 23].

Our second goal was to identify which factors of social functioning are impaired in patients with schizophrenia. To do so we examined differences between groups in those factors. Our third goal was to investigate how these social cognitive factors are related to social functioning in patients with schizophrenia and healthy matched controls. To do so, we examined relationships between factors and social-emotional functioning. We used an emotional problem-solving task and a proxy measure of social performance as proximal measures of social-emotional functioning and a quality of life assessment as a more distal measure.

We hypothesized (1) that we would find distinct factors among our social cognition and emotional experience of relationships measures, (2) that the patient sample would be more impaired on these factors than the healthy matched controls, and (3) that impairment on these factors would be related to problems in social-emotional functioning.

## 2. Methods

### 2.1. Sample Characteristics

Participants were 30 adult outpatients meeting DSM-IV [24] criteria for schizophrenia or schizoaffective disorder, as assessed by the Structured Clinical Interview (SCID) [25] by a licensed clinical psychologist, and 24 healthy controls were screened for psychiatric disorders with the Nonpatient Edition of SCID (SCID-I/NP) [26]. Patients were recruited from an urban community mental health center and were referred by their clinicians or responded to flyers posted around the center and community. Healthy control subjects were recruited through advertisements (flyers, internet posts) and selected to match as closely as possible the racial, age, and gender composition of the patient sample. After complete description of the study, subjects provided written informed consent. The Yale Human Investigation Committee approved all procedures.

Patients were clinically stable (no hospitalization, emergency room visits, homelessness, or substance abuse in the last six months). All participants were without history of epilepsy, neurological disease, brain injury, or developmental disability and were right handed, native English speakers with normal or corrected to normal vision. All patients were taking medication (see Table 1 for Chlorpromazine equivalent doses [27]). Both groups completed a clinical interview and a battery of neurocognitive and social cognitive measures administered by a licensed clinical psychologist. Symptom assessments, neurocognitive assessments, and social cognitive assessments were generally performed on different days within a week period.

### 2.2. Measures

#### 2.2.1. Symptom Assessment

The *Positive and Negative Syndrome Scale* (PANSS) [28] was used to assess current illness levels. Symptom scores were reported following the three PANSS subscales: positive, negative, and general. Interrater agreement for the PANSS in our lab was in excellent range (ICC = 0.88).

#### 2.2.2. Neurocognitive Measures

The *MATRICS Consensus Cognitive Battery *(MCCB) [29] is the gold-standard neurocognitive battery for schizophrenia research trials. It was part of the Measurement and Treatment Research to Improve Cognition in Schizophrenia Initiative (MATRICS) and 7 major separable domains of neurocognition were identified [30]: Verbal Learning and Memory, Visual Learning and Memory, Working Memory, Reasoning and Problem Solving, Speed of Processing, and Attention/Vigilance and social cognition (for each of the MCCB variables *T*-scores less than 40 reflect impairment). Because we specifically aimed at studying separately neurocognition from social cognition we excluded the Social Cognition Index score in the overall neurocognitive composite score. Therefore for this study we computed an overall neurocognitive composite score based on the average of the *T*-scores of the six neurocognitive scores.

#### 2.2.3. Social Cognitive Measures

*Emotional Perception and Processing Measures*

1. *The Social Attribution Test-Multiple Choice* (SAT-MC) version was initially created by Klin and colleagues [31, 32] and later used by Bell and colleagues [33] for schizophrenia research. It consists of a 64-second animation created by Heider and Simmel [34] in which geometric shapes enact a social drama. The animation is shown twice and then short segments are presented to accompany 19 multiple choice questions with 4 possible responses each about the actions depicted. It is scored for total number correct.
2. *The Bell-Lysaker Emotion Recognition Task* (BLERT) [35] is an affect recognition task consisting of 21 short video clips in which an actor performs one of three dialogues while portraying seven different emotions (happiness, sadness, anger, fear, surprise, disgust, and no emotion). The examinee chooses from a list the option that best reflects the affective quality portrayed. Total correct score was used (from 0 to 21).
3. *The Hinting Task* [36, 37] is a Theory of Mind measure that consists of 10 brief scenarios of an interaction between two people. One of the characters drops an obvious hint (e.g., “Jane, I'd love to wear that blue shirt, but it's very wrinkled”), and the examinee is asked what was meant. It is scored for total number correct.

*Emotional Experience Measures*

1. *Bell Object Relations Reality Testing Inventory* (BORRTI) [38] *Egocentricity and Alienation scale*: the BORRTI is a self-report measure with 90 true/false items assessing 4 dimensions of object relations and 3 dimensions of reality testing. It was developed initially for schizophrenia research and has been found to have strong psychometric properties in a wide variety of applications and to have cross-cultural validity [39].
  - The Alienation scale has been strongly associated with schizophrenia.
  - The Egocentricity scale has been associated with a more autistic understanding of others and linked with measures of social functioning [2, 23]. Higher *T*-scores on the Alienation and Egocentricity subscale indicate more impairment.
2. *The Interpersonal Reactivity Index* (IRI) [40] is a self-assessment questionnaire consisting of four seven-item subscales, each covering a separate facet of empathy. Participants score each item from a five-point scale selecting the descriptor that best suits him/her. The total scores form each element of empathy ranging from 0 to 28.
   
Two subscales measure cognitive elements of empathy.
    (a) Perspective Taking scale (IRI-PT) measures the reported tendency to adopt the psychological point of view of others in everyday life.
    (b) Fantasy Scale (IRI-FS) measures the tendency to imaginatively transpose oneself into fictional situations.
   
The second pair of subscales measure emotional aspects of empathy.
    (a) Empathic Concern (IRI-EC) assesses the tendency to experience feelings of sympathy and compassion for unfortunate others.
    (b) Personal Distress (IRI-PD) taps the tendency to experience discomfort in response to extreme distress in others. Higher scores on the IRI-PD subscale indicate more distress.

#### 2.2.4. Functional Measures

*Proxy Measure of Functional Capacity*. Social Skills Performance Assessment (SSPA) [41] is a performance-based role-play measure of social competence created for schizophrenia research. After a 1-minute practice session (which serves to acclimate the participant to the testing situation), participants initiate and maintain a conversation for three minutes in two situations: situation A is meeting a new neighbor. The participant plays the role of the tenant meeting a new neighbor (interviewer). Situation B is calling a landlord to complain about a leak that has not been fixed. The participant plays the role of a tenant calling the landlord (interviewer). The role plays are performed between an interviewer and the participant. The interviewer announces the new situation and gives the participant a written description of the situation to be performed. The interviewer's role is to reciprocate the conversation initiated by the participant using prescribed prompts as required. The overall time of the whole test is between 12 and 15 minutes approximately. For this study, all the sessions were audiotaped and scored by a rater blind to subject diagnosis from the University of California, San Diego, where the test was created. For each role play, Likert-type ratings (1 to 5) in areas of their social skills ranging from “social appropriateness” to “grooming” are made. Total scores were used in this study.

*Emotional Problem-Solving Measure. MCCB Social Cognition Index* is the seventh domain selected by the MATRICS initiative. As described above we did not include this domain with the neurocognitive measures. This domain is comprised of scores from the Mayer-Salovey-Caruso Emotional Intelligence Test (MSCEIT) [42], Emotion Management Task (Section D), and Social Management Task (Section H). The MSCEIT has recently been nominated as a candidate measure to examine emotional regulation in schizophrenia by the Social Cognition Psychometric Evaluation (SCOPE) study [12]. Respondents evaluate how effective different actions would be in achieving an outcome involving other people (e.g., how effective would calling friends or eating healthy be in making someone feel better). *T*-scores lower than 40 indicated impairment.

*Quality of Life Assessment. *Quality of life was measured using QLS [34, 43], a semistructured interview assessing various components of functioning, with items rated on a 0–6 Likert-type scale and grouped into four domains of function: interpersonal relations, intrapsychic foundations, instrumental role function, and common objects and activities. The total score ranges from 0 to 126, with higher scores indicating better function (QLS Total Score interrater agreement coefficient; ICC = 0.95).

### 2.3. Statistical Analysis

All data were analyzed using the Statistical Package for the Social Sciences (SPSS 19). Measures were checked for their distributional properties using the box-plot function of SPSS. No cases were identified as extreme outliers; therefore all data were retained. Analyses were conducted in 3 phases. First, with the combined samples, bivariate correlations of the 9 social cognitive measures were examined using correlation coefficients and afterward we conducted a principal component analysis (PCA) with the same measures. The PCA was performed following a varimax rotation to maximize orthogonality and Kaiser normalization. Factors with eigenvalues > 1 were included in the final model. Scree plots were examined to confirm factor selection [44]. The subject-to-item ratio of the PCA was of 54/9. Since the overall sample size was limited, a conservative criterion was used to determine final factor structure. We retained items only with high communalities (0.5 and above) and without cross-loadings and retained only factors with at least two items [45]. Secondly, we performed one-way ANOVAs comparing the two groups on the derived social cognitive factors. Finally, we correlated the derived social cognitive factors with the MCCB neurocognitive composite score, symptoms, and the measures of social functioning, firstly with both groups together and secondly with groups separately. Group differences between correlations were calculated using Fisher *r*-to-*z* transformation.

## 3. Results

### 3.1. Sample Characteristics and Descriptive Statistics (Table 1)

The patient sample was chronically ill and without gender predominance. The healthy control group was selected to match the demographic characteristics of the patient sample and this matching effort was mostly successful. Neither gender nor ethnicity differed between groups. Although groups differed in years of education, parental education did not differ. The mean age in the groups differed; however, both samples were comprised of middle-aged adults.

### 3.2. Group Comparisons (Table 2)

Healthy controls scored significantly higher than patients on the SAT-MC, BLERT, Hinting Task, MSCEIT, SSPA the IRI-PT, QLS Total Score, and the neurocognitive composite of the MATRICS. In addition, patients showed significantly greater impairment on the BORRTI Egocentricity and Alienation subscales and reported greater distress on the IRI-PD subscale.

### 3.3. Correlation Analysis (Table 3)

The Hinting Task, BLERT, and SAT-MC were strongly intercorrelated. Intercorrelations within IRI subscales were minimal except with IRI-EC which correlated with IRI-PT and IRI-FS. The subscales IRI-PT and IRI-FS did not correlate with each other, and IRI-PD revealed an inverse correlation with IRI-PT. The Hinting Task correlated with the IRI-FS and the SAT with IRI-EC and IRI-FS. BORRTI Alienation and Egocentricity scores correlated with each other and inversely correlated with IRI-PT. Additionally, BORRTI Alienation correlated with the IRI-PD.

### 3.4. Principal Components Analysis of Social Cognition Measures and Group Comparisons (Tables 4 and 5 and Figure 1)

In the PCA of the 9 social cognitive subtasks (derived from five main measures), the eigenvalue-greater-than-one criterion and scree plot converged in a 3-factor solution after varimax rotation, which accounted for 62.61% of total variance. Factor 1, named Interpersonal Discomfort (ID), represented 29.79% of the total variance and included the IRI-PD and the BORRTI Egocentricity and Alienation measures. Factor 2, named Basic Social Cognition (BSC), included the BLERT, SAT, and Hinting Task and accounted for 17.49% of the total variance. Finally, factor 3, named Empathy (Em), was comprised of the IRI-PR, IRI-EC, and IRI-FS and accounted for 15.32% of the total variance. Group comparisons revealed significant differences in Interpersonal Discomfort (*F*_(1,52)_ = 30.412, *P* < 0.01) and Basic Social Cognition (*F*_(1,52)_ = 10.927, *P* < 0.01). However, no differences were found for Empathy (*F*_(1,52)_ = 0.614, *P* = 0.437).

### 3.5. Relationship of Factors to Symptoms, Neurocognition, and Social Functioning (Table 6)

Factors showed no significant correlations with negative, positive, or general symptoms, assessed by the PANSS. Correlations between the social cognitive factors and the MCCB neurocognitive composite scores revealed highly significant correlations between Basic Social Cognition and MCCB neurocognitive composite scores for the whole sample and for patients and healthy controls separately. While the sample as a whole showed a significant inverse correlation between MCCB neurocognitive composite and Interpersonal Discomfort, this significant relationship was not present in either group separately. MCCB neurocognitive composite had the strongest correlation with Basic Social Cognition for the whole sample and had significant relationships with each group separately.

Performance on the SSPA was significantly and highly correlated with Basic Social Cognition in both groups together. This correlation remained significant in the patient group alone but not in the controls, although the difference itself between groups was not significant. Highly significant inverse correlations were found between the SSPA and Interpersonal Discomfort in both samples together and in just the healthy controls. Fisher *r*-to-*z* comparison between group correlations indicated that groups differed in this correlation, confirming the inverse relationship in the healthy control group but not in patients.

Similar to results obtained with the SSPA, the QLS revealed a strong negative correlation with Interpersonal Discomfort, in both samples together and just alone in the healthy controls. Group correlations differed significantly in this relationship suggesting that just the healthy controls show this inverse relation. Additionally, Basic Social Cognition correlated with the QLS in both samples together, but no differences were found between groups in this relationship.

Correlations with the MSCEIT-ME and the factors revealed a highly significant correlation with Empathy, in groups together and separately, and a moderate inverse correlation with Interpersonal Discomfort for the sample as a whole. This significant correlation was not found in the groups separately and no differences were found between groups.

## 4. Discussion

Analysis of measures of social cognition and emotional experience in patients with schizophrenia and a healthy control sample identified three distinct factors. Patients showed abnormalities on two of the factors but on not the third, providing new insight into the nature of social cognition dysfunction in schizophrenia. Differential associations between these factors and proxy and distal measures of functioning provide additional information about the nature of social dysfunction in patients, which may be of potential relevance for treatment development.

Factor analysis of scores from specifically selected tasks (emotion recognition (BLERT), ToM (Hinting Task), Social Attribution (SAT-MC), four empathy subscales (IRI), and two subscales of Self-Experience of Relatedness (BORRTI)), yielded three factors that explained more than 62% of the variance and which we named Interpersonal Discomfort, Basic Social Cognition, and Empathy. When we compared groups on these factors, we discovered that patients with schizophrenia were significantly more impaired than healthy controls on Interpersonal Discomfort and Basic Social Cognition but not on Empathy. This finding suggests that Empathy, which is comprised of Perspective Taking, Empathic Concern, and Empathic Fantasy, may be preserved in schizophrenia. In other words, patients may retain the capacity to care about others and to imagine the pain others may go through just as well as healthy individuals. Their impaired social functioning may come from social cognitive deficits represented by the other two factors that interfere with their empathic capacity. They may fail to correctly read the emotions of others (Basic Social Cognition) so that they do not know that the other person may be in distress, and when they do correctly perceive the other person's emotions, the high degree of Interpersonal Discomfort they experience may make them want to avoid the interaction. In other words, impairments in Basic Social Cognition and high Interpersonal Discomfort are barriers to the using of their empathic concern, even though they still retain that capacity.

Empathy is a multidimensional construct that captures both cognitive perspective taking abilities and emotional experiences of empathy [46]. The IRI was specifically designed to target the emotional and cognitive features of empathy. Even though the Perspective Taking subscale of the IRI was significantly different between groups, the fact that it loaded in the Empathy factor and this factor did not differ between groups could suggest that cognitive perspective abilities are needed for adequate levels of empathy.

Regarding the relationship between clinical symptoms and social cognition, our findings suggest that they are not significantly related and therefore social cognition may represent a distinct and independent domain of psychopathology. These findings are consistent with the findings from prior studies particularly in regard to social cognitive abilities and negative symptoms [9, 47, 48], but also found with positive symptoms [49].

The Interpersonal Discomfort factor was comprised of the Personal Distress subscale of the IRI and the Alienation and Egocentricity scores from the BORRTI. We chose the descriptive term “Interpersonal Discomfort” because it is a bridging construct between the Personal Distress captured by the IRI and the alienation and withdrawal into an egocentric self-protective view of relatedness that is measured by Alienation and Egocentricity on the BORRTI. Both the IRI and BORRTI are self-report measures of the Self-Experience of Relatedness, and one feature of that relatedness that has emerged in factor analytic studies of these scales has been the discomfort that interpersonal relationships can bring. The significantly higher ratings in patients than controls in Personal Distress [13, 50, 51] at the distress of others convey a view of social isolation in schizophrenia at odds with the idea that it is secondary to negative symptoms or that it indicates deficits in social and emotional sensitivity.

The Interpersonal Distress factor showed a strong negative correlation with social competence on the SSPA and QLS in healthy controls but not in patients. These correlations suggest that emotional and social stress influence performance and real-world functioning in even healthy individuals. The absence of these understandable correlations in patients requires explanation. We propose that the lack of correlation with QLS in the patient group is because there are other more powerful factors limiting patient quality of life such as social stigma and unemployment that obscured the role of social cognition. In our healthy control sample, variation in quality of life may have been less influenced by these factors and therefore more of the variance was explained by social cognition.

The second factor, Basic Social Cognition, was comprised of performance on the BLERT, a measure of the ability to identify emotion expressed in facial displays and quality of voice, and performance on the social attribution task, SAT-MC, and the ToM task (Hinting task) which assess ability to deduce or imagine what others are feeling and thinking, respectively. Patients had lower scores on this factor than healthy controls, consistent with an established literature [52]. This factor was not correlated with the SSPA proxy measure of functional capacity in healthy controls, presumably because all had minimum necessary competence. In patients, however, Basic Social Cognition was robustly correlated with the SSPA measure of social function, suggesting that social role-play performance is dependent on basic social cognitive processes like emotion recognition, social attribution, and Theory of Mind.

A recent study by Green and colleagues [53], in which they used structural equation modeling to evaluate models of pathways to functional outcome starting with early visual perception, found that their proxy measure of functional capacity did not add any variance beyond the one explained by their unique social cognition factor. They suggested that functional capacity should be considered as part of a general ability factor in conjunction with social cognition and neurocognition. In our study, patients but not matched healthy controls revealed a strong correlation between basic social cognition and functional capacity. This finding suggests that perhaps the “general ability factor” described by Green and colleagues [53] could be uniquely related to the schizophrenia population and not to healthy controls.

In both healthy controls and patients, Basic Social Cognition was correlated with general cognition as measured in the MATRICS battery. This finding in patients is also well established in the literature and justifies treatment approaches that begin with efforts to restore general cognitive abilities [54, 55].

The third factor, Empathy, was comprised of the three subscales of the IRI other than Personal Distress: Empathic Concern, Perspective Taking, and Fantasy. Here, quite remarkably, there was no difference between patients and controls. Moreover, in both patients and controls the Empathy factor was robustly correlated with the MSCEIT measure of emotion management. Those individuals who are better at managing and regulating their emotions are also more able to experience empathic concern, take the perspective of others, and have the ability to imaginatively transpose themselves into the experience of another person. Our findings are in agreement with previously published studies regarding the IRI subscales in schizophrenia showing impaired perspective taking [13, 51, 56–59] and impaired personal distress [13, 51, 56, 57, 60].

Strikingly, Empathy was not related to social capacity and competence suggesting that empathic skills alone do not enable a person to perform and function properly in a social situation. Real life functioning as measured by the QLS was not related to Empathy as well. Thus, it seems that empathic skills may not be sufficient to perform and operate effectively in social situations as indicated by the SSPA. However, in order to effectively manage, regulate, and solve difficult emotional and social problems for oneself and for others, empathy seems to be basic, as suggested by the correlation with the MSCEIT. Additionally, Empathy was the only factor not related to neurocognition. This independence from neurocognition suggests that it may be a preserved feature in schizophrenia, even in neurocognitively impaired individuals and that it could be a potential social cognitive strength to be called upon during rehabilitation.

Our results are in agreement with Mancuso and colleagues, in which they also did not find any relationship between their social cognitive measures and their outcome factors and negative symptoms [9]. However, their Hostile Attributional Style factor uniquely correlated with positive symptoms and depression. All the measures that loaded in that factor were part of the Ambiguous Intentions Hostility Questionnaire (AIHQ [61]), which has items which may be closer to symptoms such as suspiciousness than to social cognition. Mancuso and colleagues' factor structure followed what they stated as information processing levels structure. We believe that our structure is not defined by information processing stages of the emotional and social stimuli but by perceptual and unique experiential stages of processing, a result which is different but may not be contradictory to theirs. Our factor structure produced the Basic Social Cognition factor which covered foundational social perception abilities and is similar to their lower-level social cue detection factor. Regarding their higher-level inferential and regulatory processing factor, we did not find a factor in that regard since we did not include emotional regulatory and second-order Theory of Mind tests.

Two of the three factors comprised measures of emotional experience. We believe that this finding can be due to the fact that more measures on this domain were used in the final factor analysis, but we also wanted to explore whether these measures would overlap with emotional perception measures. Interestingly, they did not overlap. For example, one could have expected that the Perspective Taking scale of the IRI would have been weighted on the same factor as the ToM scale, since this scale correlates highly with cognitive empathy measures [62]. Emotional responses are comprised of multiple components (e.g., emotional experience, expression, and physiology) [19], and our findings support current evidence suggesting that the emotional and social cognitive deficits seen in schizophrenia may be contingent on the type and stage of emotional response that is being evaluated [17, 19, 20]. Our results provide additional support for the importance of including measures of experience and perception in schizophrenia research. Future studies should include expressive, physiological, and other functional measures in their component analysis to target as many components of the emotional response as possible.

While this study did not involve an intervention which aimed at improving social cognition, findings may be useful in considering what types of social cognitive impairments to target in an intervention and what may be important to be measured as outcome variables. Data reduction is a significant scientific concern in such studies and our findings may be useful for that purpose. We also think our finding of preserved empathy suggests that if basic social cognition can be improved and personal distress can be reduced, the patient's capacity for empathic concern could naturally emerge as a social cognitive strength. We hope that this study will promote further investigation into empathy in schizophrenia so that the current existent gap in the literature can be filled.

There are several limitations to the current study. Although we selected our measures because of their specificity at targeting different social cognitive constructs, these measures may not have included some important social cognitive domains. Also, some of our measures may have overlapped, thus putting too much emphasis on one domain of social cognition. Sample sizes were relatively small but large enough for the PCA's and comparisons between groups; the exploratory regression analyses should be viewed as preliminary findings that will need substantial verification in larger samples. Patients in this sample were stable outpatients who had lived with psychiatric illness for more than two decades. It is not known whether these findings generalize to younger samples who have not been subjected for so long to the social consequences of psychiatric illness. Finally, while combining samples broadened the range of functioning represented in this study, it presented the complication that group membership and neurocognitive status were almost interchangeable. However, the independence of Empathy from group membership is a discovery that was only possible by combining the groups and offers a potentially valuable new consideration in understanding resilience in schizophrenia.

## 5. Conclusions

Three distinct factors were identified with different relationships to neurocognition and social functioning. Empathic concern may be a preserved capacity in patients and it is an ability that is related to emotion management. However, while patients may have a preserved ability for empathic concern, they may be hampered by the emotional discomfort they feel in relation to others and this emotional discomfort along with impairments in basic social cognition impairs their social function. These findings imply that social cognitive interventions should emphasize improving or compensating for Basic Social Cognitive processing deficits, managing Interpersonal Discomfort, and utilizing preserved capacity for empathy as a potential strength in forming and maintaining relationships.

## Figures and Tables

**Figure 1:**
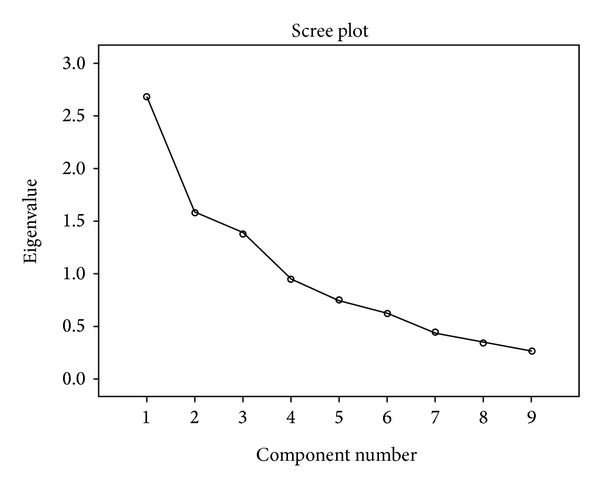
Scree plot.

**Table 1:** Characteristics for demographic, functional, and clinical measures.

	Patients (*n* = 30)	Healthy Controls (*n* = 24)	*F*/Chi square	Degrees of freedom	*P*
Age (mean, SD)	46.47 (8.22)	39.79 (8.87)	8.195	(1, 52) 53	<0.01
Gender (*n*, %)			1.344	1	ns
Male	14 (46.7%)	15 (62.5%)			
Female	16 (53.3%)	9 (37.5%)			
Ethnicity (*n*, %)			0.627	1	ns
African-American	17 (56.7%)	11 (45.8%)			
Caucasian	13 (43.3%)	13 (54.2%)			
Education years (mean, SD)	13.13 (1.78)	16.25 (2.54)	28.065	(1, 52) 53	<0.01
Parental education years (mean, SD)*	12.65 (5.11)	14.26 (2.70)	1.819	(1, 47) 48	ns
Age at illness onset (mean, SD)	24. 27 (8.05)	NA			
Illness duration, in years (mean, SD)	22.20 (10.30)	NA			
GAF (mean, SD)***	52.15 (15.02)	NA			
Schizophrenia diagnosis (*n*, %)					
Paranoid	17 (56.7%)	NA			
Undifferentiated	3 (10.0%)	NA			
Schizoaffective	13 (33.3%)	NA			
PANSS (mean, SD)					
Positive	18.93 (6.52)	NA			
Negative	17.00 (6.10)	NA			
General	30.17 (10.02)	NA			
Chlorpromazine equivalents (md/day)^†^	654.03 (659.21)	NA			

Abbreviations: GAF: Global Assessment of Functioning; PANSS: Positive and Negative Syndrome Scale.

*includes 26 patients and 23 healthy controls.

**includes 22 healthy controls.

***includes 26 patients.

^†^includes 28 patients.

**Table 2:** Social cognitive and neurocognitive measures.

	Patients (n = 30) mean, SD	Healthy controls (n = 24) mean, SD	*F* value	Degrees of freedom	P*
Neurocognition					
Neurocognitive MCCB^§^	39.58 (7.17)	52.05 (8.14)	35.76	(1, 52) 53	<0.01
Emotional perception and processing					
Social Attribution (SAT-MC)	11.70 (4.56)	15.21 (3.23)	10.13	(1, 52) 53	<0.01
Emotion Recognition Test (BLERT)	13.67 (3.48)	16.46 (2.67)	10.50	(1, 52) 53	<0.01
Theory of Mind (The Hinting Task)	16.80 (2.73)	19.13 (0.68)	16.48	(1, 52) 53	<0.01
Emotional Experience					
Self-Experience of Relatedness (BORRTI)					
Egocentricity	59.77 (12.55)	44.88 (8.48)	24.72	(1, 52) 53	<0.01
Alienation	56.60 (10.15)	43.00 (8.71)	27.09	(1, 52) 53	<0.01
Empathy (IRI)					
Perspective Taking	16.13 (4.97)	19.08 (4.02)	5.54	(1, 52) 53	<0.05
Fantasy	13.37 (6.71)	13.79 (5.40)	0.06	(1, 52) 53	ns
Empathic Concern	18.53 (5.29)	20.00 (4.85)	1.10	(1, 52) 53	ns
Personal Distress	11.80 (4.64)	9.21 (4.37)	4.37	(1, 52) 53	<0.05
Social Functioning					
Functional Capacity (SSPA)	62.00 (11.10)	75.08 (8.07)	23.41	(1, 52) 53	<0.01
Emotional problem Solving (MSCEIT-ME)	38.53 (13.59)	50.21 (12.00)	10.90	(1, 52) 53	<0.01
Quality of Life Scale (QLS)	61.07 (19.50)	112.45 (13.97)	110.81	(1, 50) 51	<0.01

Note: *Social Cognitive Measures*: SAT-MC: Social Attribution Task-Multiple Choice, correct score; BLERT: Bell-Lysaker Emotion Recognition Test, correct score; Hinting: The Hinting Task, total score; BORRTI Egocentricity: Bell Object Relations Reality Testing Inventory, Egocentricity subscale and Alienation subscale; IRI: Interpersonal Reactivity Index; *Social Functioning:* MSCEIT-ME: Mayer-Salovey-Caruso Emotional Intelligence Test-Managing Emotions Branch, scaled score; overall subscores; SSPA: Social Skills Performance Assessment, overall correct score; QLS: Quality of Life Scale.

*Patient and control group differences by two-sided one-way ANOVA.

^§^Neurocognitive MCCB: Neurocognitive Composite Score of the MATRICS Consensus Cognitive Battery: the overall neurocognitive composite score excludes the Social Cognition Index since it is a social cognition measure, based on the average of *T*-scores of the six neurocognitive domains.

**Table 3:** Correlations among the social cognitive measures in both groups.

Pearson correlation coefficients	Theory of Mind	Emotion Recognition	Social Attribution	Empathy Perspective Taking	Empathy Empathic Concern	Empathy Personal Distress	Empathy Fantasy	Self-relatedness, Alienation	Self-relatedness, Egocentricity
Theory of Mind	1								
Emotion Recognition	0.421**	1							
Social Attribution	0.370**	0.384**	1						
Empathy Perspective Taking	−0.05	0.023	0.253	1					
Empathy Empathic Concern	0.181	−0.052	0.292*	0.376**	1				
Empathy Personal Distress	−0.184	−0.383**	−0.165	−0.301*	−0.149	1			
Empathy Fantasy	0.281*	0.241	0.322*	0.24	0.407**	0.039	1		
Self-Relatedness, Alienation	−0.211	−0.217	−0.044	−0.313*	−0.168	0.314*	0.119	1	
Self-Relatedness, Egocentricity	−0.111	−0.255	−0.135	−0.276*	−0.068	0.098	−0.018	0.552**	1

**Correlation is significant at the 0.01 level (2-tailed).

*Correlation is significant at the 0.05 level (2-tailed).

**Table 4:** Eigenvalues extracted by the principal component analysis.

Component	Initial eigenvalues		
	Total	% of variance	Cumulative %
1	2.681	29.794	29.794
2	1.575	17.499	47.292
3	1.379	15.325	62.618
4	0.948	10.530	73.148
5	0.751	8.346	81.495
6	0.622	6.906	88.401
7	0.443	4.920	93.321
8	0.338	3.756	97.077
9	0.263	2.923	100.000

**Table 5:** Factor loadings of the social cognitive measures.

	Component		
	Interpersonal Discomfort	Basic Social Cognition	Empathy
Emotion Recognition test (BLERT)	−0.073	**0.773**	0.083
Social Attribution (SAT-MC)	−0.281	**0.81**	−0.107
Theory of Mind (The Hinting Task)	−0.042	**0.603**	0.436
Empathy (IRI)			
Perspective Taking	−0.491	−0.148	**0.666**
Empathic Concern (EC)	−0.096	0.03	**0.822**
Personal Distress	**0.521**	−0.309	−0.059
Fantasy	0.245	0.399	**0.67**
Self-Experience of Relatedness (BORRTI)			
Alienation	**0.859**	−0.058	−0.026
Egocentricity	**0.736**	−0.086	−0.046

Note: BLERT: Bell-Lysaker Emotion Recognition Test; SAT-MC: Social Attribution Task-Multiple Choice, correct score; The Hinting Task; IRI: Interpersonal Reactivity Index; BORRTI: Bell Object Relations Reality Testing Inventory.

Factor loadings higher than 0.5 are in bold.

**Table 6:** Correlations between neurocognition, social capacity, quality of life, and emotional problem solving with the factors.

Social cognitive factors	Patients and healthy controls combined	Patients	Healthy controls	*P* ^†^
Neurocognitive (MCCB)				
Interpersonal Discomfort	−0.421**	0.011	−0.198	ns
Basic Social Cognition	0.591**	0.431*	0.659**	ns
Empathy	0.021	−0.14	0.037	ns
Quality of Life (QLS)				
Interpersonal Discomfort	−0.568**	−0.045	−0.588**	<0.05
Basic Social Cognition	0.369**	−0.031	0.256	ns
Empathy	0.143	0.151	0.116	ns
Functional capacity (SSPA)				
Interpersonal Discomfort	−0.407**	0.037	−0.553**	<0.05
Basic Social Cognition	0.503**	0.411*	0.195	ns
Empathy	0.199	0.092	0.327	ns
Emotional problem solving (MSCEIT-ME)				
Interpersonal Discomfort	−0.293*	0.044	−0.319	ns
Basic Social Cognition	0.21	0.066	−0.013	ns
Empathy	0.572**	0.565**	0.613**	ns

**Correlation is significant at the 0.01 level (2-tailed).

*Correlation is significant at the 0.05 level (2-tailed).

^†^Difference between patients' and healthy controls' correlation by using Fisher r-to-z transformation.

Note: MCCB: MATRICS Consensus Cognitive Battery; QLS: Quality of Life; SSPA: Social Skills Performance Assessment; MSCEIT-ME: Mayer-Salovey-Caruso Emotional Intelligence Test-Managing Emotions Branch.

## Abbreviations

PCA: Principal components analysis
ToM: Theory of Mind
BLERT: Bell-Lysaker Emotion Recognition Test
BORRTI: Bell Object Relations Reality Testing Inventory
MSCEIT: Mayer-Salovey-Caruso Emotional Intelligence Test
QLS: Quality of Life Scale
SAT-MC: Social Attribution Test-Multiple Choice
MCCB: MATRICS Consensus Cognitive Battery
IRI: Interpersonal Reactivity Index
PT: Perspective Taking scale
FS: Fantasy Scale
EC: Empathic Concern
PD: Personal Distress
PANSS: Positive and Negative Syndrome Scale.
